# Network dynamics of international food waste trade: Evidence from the COVID-19 disruption

**DOI:** 10.1016/j.crfs.2026.101336

**Published:** 2026-02-02

**Authors:** Navid Mohammadi, Mehrdad Maghsoudi

**Affiliations:** a College of Management, University of Tehran, Tehran, Iran; b Management and Accounting Faculty, Shahid Beheshti University, Tehran, Iran

**Keywords:** Food waste management, Network analysis, International trade, COVID-19, Circular economy

## Abstract

This paper presents a comprehensive network analysis of global food waste management trade patterns before and after the COVID-19 pandemic. Using World Bank export data from 2015 to 2024, we construct directed, weighted networks to examine structural transformations in international trade relationships. Our analysis reveals three key findings: first, while network density and connectivity remained stable, we observed a 9% decrease in average trade volume post-pandemic, suggesting supply chain recalibration. Second, centrality metrics demonstrate the continued dominance of the United States and European nations, though with emerging economies like India and Brazil forming new strategic alliances. Third, community detection algorithms identify a significant reorganization of trade blocs, particularly the formation of a distinct Emerging Economies cluster separate from traditional Western networks. These findings contribute to ongoing discussions about resilience in global food systems, offering empirical evidence of how major disruptions reshape trade relationships while maintaining core structural integrity. The study provides policymakers with quantitative insights for developing more robust food waste management strategies within the circular economy framework.

## Highlights

• **First network analysis** comparing global food waste trade structures before and after COVID-19.
• Reveals **9% decline in trade volumes** despite preserved network connectivity.
• Documents **formation of new trade blocs** among emerging economies.
• Provides **policy-ready insights** for strengthening circular waste systems.
• Combines **macro-scale network metrics** with **micro-level community detection.**

## 1 introduction

In an era where global food systems face unprecedented challenges, the stark reality of food loss and waste (FLW) stands as a testament to the inefficiencies that plague our modern world. Each year, approximately one-third of all food produced for human consumption - a staggering 1.3 billion tons - is lost or wasted, creating ripples of consequences that extend far beyond mere economic losses. This crisis, while daunting in its scale, represents not just a challenge but an opportunity for transformative change in how we manage and utilize our food resources. The economic ramifications of this global predicament are profound, with direct economic losses reaching USD 1 trillion annually (Al-Obadi et al., 2022; Zan et al., 2022). When considering the broader spectrum of economic, environmental, and social impacts, the total burden on the global economy escalates to an estimated USD 2.6 trillion. Yet, these figures tell only part of the story. In a world where approximately 690 million people face daily hunger, the paradox of massive food waste becomes even more poignant, highlighting the urgent need for systematic change in our approach to food waste management (Bernstad and la Cour Jansen, 2012; Närvänen et al., 2020).

The environmental footprint of this inefficiency casts an even longer shadow. Food waste has emerged as a significant contributor to global greenhouse gas emissions, accounting for 8–10% of total emissions. This environmental burden is further amplified by the fact that approximately 1.4 billion hectares of land - an area larger than China - is utilized annually to produce food that will never reach a human plate (Huang et al., 2021; Rashid and Shahzad, 2021). The carbon footprint of food wastage, estimated at 3.3 billion tons of CO2 equivalent per year, serves as a stark reminder of the environmental cost of our current practices. In response to these challenges, the international community has mobilized through various initiatives and policy frameworks. The United Nations' Sustainable Development Goal Target 12.3, which aims to halve per capita global food waste at retail and consumer levels by 2030, has catalyzed a wave of national and regional responses. From South Korea's innovative Volume-Based Waste Fee system to the European Union's ambitious Circular Economy Action Plan, countries worldwide are demonstrating that effective intervention strategies are possible and impactful (Chinda and Thay, 2022; Dalke et al., 2021).

The complexity of these interconnected challenges demands sophisticated analytical approaches to understand and address global food waste management systems. Network analysis has emerged as a powerful tool in this context, offering unique insights into the structural properties and dynamics of global food systems (Coşkuner and Sogah, 2023; Lin et al., 2023; Liu and Chen, 2023). This methodological approach has proven particularly valuable in analyzing trade flows and identifying key players in international markets, providing a framework for understanding the complex web of relationships that characterize global food waste management (Dalke et al., 2021; Deng et al., 2021; Grassi et al., 2021; Herman, 2022).

Our study leverages this analytical power by applying network analysis techniques to World Bank export data, aiming to evaluate cross-country performance in food waste management. This approach allows us to uncover patterns and relationships that might otherwise remain hidden, offering fresh perspectives on successful management practices and areas for improvement. By examining the intricate network of international trade relationships in food waste management, we seek to identify key actors, successful strategies, and potential opportunities for enhanced cooperation and efficiency. The significance of this research extends beyond academic inquiry. Our findings have the potential to inform policy decisions, guide international cooperation efforts, and contribute to the development of more effective food waste management strategies. Through careful analysis of trade patterns and relationships, we aim to provide actionable insights that can help countries optimize their food waste management practices and contribute to global sustainability goals.

As we delve deeper into this analysis, our research not only bridges the gap between theoretical frameworks and practical implementation but also offers a quantitative foundation for evaluating and improving global food waste management systems. In doing so, we contribute to the broader goal of creating more sustainable and efficient food systems worldwide, addressing one of the most pressing challenges of our time through the lens of network analysis and international trade relationships.

The remainder of this paper is structured as follows. First, we review the literature on food waste management, international trade patterns in this domain, and related works. We then present our methodological framework, which applies network analysis techniques to World Bank export data. Following this, we present and discuss our empirical findings, highlighting key patterns in global food waste management networks. The paper concludes with implications for policy and practice, along with recommendations for future research.

## 2 Literature review

### 2.1 Food waste management

Food waste management has evolved significantly over the past decades, transforming from simple disposal methods to sophisticated, integrated approaches that reflect our growing understanding of environmental and social responsibilities. The earliest recorded waste management practices date back to 3000 B.C. in Knossos, Crete, where rudimentary landfills were used for waste disposal. However, the modern conceptualization of food waste management emerged during the late 19th and early 20th centuries, driven by rapid urbanization and industrialization that necessitated more organized and systematic approaches to handling increasing volumes of waste.

In contemporary discourse, food waste management encompasses a complex hierarchy of strategies and interventions, fundamentally guided by the principle of waste minimization and resource optimization. The widely adopted waste hierarchy framework prioritizes prevention at its apex, followed by reduction, reuse, recycling, and recovery, with disposal considered the least favorable option. This hierarchical approach has been particularly influential in shaping policy frameworks, such as the European Union's Waste Framework Directive, which has made these principles legally binding. The framework's effectiveness is evidenced by current statistics indicating that approximately 19% of total food production is wasted at the household level globally, highlighting both the challenge's magnitude and the potential for improvement through systematic management approaches.

The evolution of food waste management has been particularly marked by the emergence of circular economy principles, which have revolutionized traditional linear “take-make-dispose” models. This paradigm shift has introduced innovative approaches such as waste valorization, closed-loop systems, and reverse logistics, all aimed at maximizing resource efficiency and minimizing environmental impact. These developments have been accompanied by sophisticated technological solutions and management strategies, including the implementation of AI-driven prediction systems for food spoilage and advanced supply chain optimization techniques. The integration of these approaches has created a more comprehensive and nuanced understanding of food waste management, moving beyond simple disposal solutions to encompass the entire food value chain, from production to consumption.

### 2.2 Food waste international trade

The international trade of food waste has emerged as a critical component in the global food system, characterized by complex patterns of exchange and intricate regulatory frameworks that govern cross-border movements. The evolution of food waste trade networks has been particularly notable from 1992 to 2018, during which period globalization and changing patterns of production and consumption have significantly reshaped traditional trade relationships. This transformation has been driven by the growing recognition of food waste as both an environmental challenge and an economic opportunity, especially in the context of feeding a projected global population of nine billion by 2050.

The economic dimensions of international food waste trade are substantial, with global food waste resulting in estimated annual losses of USD 940 billion. In developed economies like the United States, these losses amount to approximately USD 218 billion annually, representing 1.3% of the country's GDP. The trade patterns exhibit distinct characteristics between developed and developing nations, with developing countries experiencing significant losses primarily during production and distribution phases due to infrastructural limitations, while developed nations face greater challenges at the consumer level. This dichotomy has created unique trade dynamics, where resource efficiency and waste management capabilities significantly influence trade flows and economic outcomes.

The regulatory landscape governing international food waste trade is multifaceted, encompassing various international agreements and frameworks. The World Trade Organization provides the foundational structure through its Agreement on Agriculture, which establishes crucial guidelines for market access, domestic support, and export competition. These regulations are complemented by regional and bilateral free trade agreements that increasingly incorporate sustainability provisions, as exemplified by the EU-Japan Economic Partnership Agreement. Furthermore, international organizations such as the United Nations and World Bank play pivotal roles in facilitating and monitoring these trade relationships, providing technical assistance and financial support to ensure compliance with international standards. This regulatory framework has been particularly influential in shaping how countries approach food waste management within the context of international trade, leading to the development of more sophisticated and environmentally conscious trading practices.

The economic viability of food waste trade is further enhanced by innovative practices and technological advancements. For instance, the implementation of circular economy principles in food waste management has demonstrated potential returns of up to 14-fold on investment for food-related businesses. These developments have been accompanied by the emergence of successful international partnerships and trading relationships, exemplified by various initiatives that connect surplus food sources with markets and communities in need across borders. Such innovations in trade practices not only address the environmental implications of food waste but also create new economic opportunities within the global food system.

### 2.3 related works

The application of network analysis to international trade and resource management has garnered significant scholarly attention in recent years, with researchers employing diverse methodological approaches to understand complex trade relationships. A critical examination of recent literature reveals both notable advances and persistent limitations in our understanding of these intricate systems. The pioneering work by Pacini et al. (2021) on plastic scrap trade networks demonstrated the dominance of EU and North American countries while highlighting a systematic underrepresentation of developing regions, establishing a foundational framework for analyzing resource flow inequalities in global trade networks.

Subsequent methodological innovations emerged through the integration of more sophisticated analytical tools. Herman (2022) introduced a novel combination of gravity models with exponential random graph models (ERGMs), demonstrating how network dependencies significantly influence trade flows. This methodological advancement was further developed by Sajedianfard et al. (2021), who addressed the critical issue of missing data in trade networks through innovative reconstruction methods. Their work demonstrated the robustness of network analysis approaches while highlighting the importance of data quality in trade studies.

Recent studies focusing on specific commodities have provided deeper insights into trade network dynamics. Zheng et al. (2022) analyzed kaolin trade patterns across 120 countries, demonstrating increasing regionalization trends and the growing influence of developing economies. Similarly, Deng et al. (2021) examined virtual water trade among major countries, revealing increasing network density in primary industries and highlighting infrastructure limitations in developing regions. These commodity-specific analyses were complemented by Grassi et al. (2021), who developed a multi-attribute community detection approach that revealed distinct trading communities and power distributions within international trade networks.

The evolution of analytical approaches has been particularly evident in food trade studies. Wang and Dai (2021) documented the transformation from unipolar to multipolar trade structures between 1992 and 2018, while Ji et al. (2024) revealed how disruptions affecting even 5% of nations could destabilize the entire food trade network. These findings gained additional relevance through Lin et al. (2023) analysis of the Russia-Ukraine conflict's impact on global food security, demonstrating how regional conflicts can cascade through trade networks to affect global food systems. Methodological innovations have continued to emerge, with Liu and Chen (2023) introducing dynamic network modeling using artificial neural networks for enterprise-level analysis. Ma et al. (2021) extended network analysis to examine ammonia emissions in trade relationships, while Coşkuner and Sogah (2023) developed an augmented gravity model incorporating social network analysis to better understand export performance across 51 countries. The summary of related works has been provided in Table 1.

**Table 1:** Related works.

Author (year)	Title	Methodology	Data Coverage	Key Findings
Pacini et al. (2021)	Network analysis of plastic scrap trade	Network centrality analysis	Global trade data (2018); 180+ countries	EU and North America dominate flows; developing regions underrepresented
Herman (2022)	Modeling complex network patterns in international trade	Gravity models + ERGMs	Bilateral trade data from 150 countries (2010–2020)	Network dependencies significantly influence trade flows
Zheng et al. (2022)	Analysis on the evolution characteristics of kaolin international trade pattern based on complex networks	Complex network model	Global data (2000–2020); 120 countries	Increasing regionalization; developing countries lack pricing power
Grassi et al. (2021)	Multi-Attribute Community Detection in International Trade Network	Community detection, centrality measures	160 countries (2015–2020)	Distinct trade communities identified
Sajedianfard et al. (2021)	Quantitative analysis of trade networks: data and robustness	Network reconstruction methods	UN Database, 10 commodity groups	Improved methods for handling missing trade data
Deng et al. (2021)	Social network analysis of virtual water trade among major countries in the world	Multi-region input-output model	19 G20 countries (2006–2015)	Dense networks in primary industries
Liu and Chen (2023)	An empirical analysis of dynamic network model of international trade by using enterprise sample simulation and improved ANN algorithm	Neural network algorithms	Enterprise-level data from 100 companies	Improved prediction accuracy for trade patterns
Wang and Dai (2021)	Evolution of Global Food Trade Patterns and Its Implications for Food Security Based on Complex Network Analysis	Complex network analysis	Global food trade (1992–2018)	Shift from unipolar to multipolar structure
Ji et al. (2024)	The structure, dynamics, and vulnerability of the global food trade network	Scale-free network analysis	180 countries (2000–2019)	System vulnerable to disruptions
Ma et al. (2021)	Mitigation potential of global ammonia emissions and related health impacts in the trade network	Network analysis of emissions	Global trade data (2012)	Trade-related emissions cause significant health impacts
Lin et al. (2023)	The impact of Russia-Ukraine conflict on global food security	General equilibrium model	51 countries (2021–2022)	Significant disruption to global food security
Coşkuner and Sogah (2023)	Augmented Gravity Model of Trade with Social Network Analysis	Gravity model with SNA	51 countries (41 years)	GDP and trade openness influence export performance

The critical analysis of existing literature reveals several significant gaps in our current understanding of trade networks. The most pressing limitation lies in the methodological realm, where current approaches largely fail to integrate qualitative factors such as policy changes, cultural influences, and informal trade relationships. This shortcoming is particularly evident in studies attempting to analyze developing regions, where data scarcity and reporting inconsistencies create substantial blind spots in our understanding of global trade patterns. Furthermore, the predominant focus on single commodity networks has limited our ability to understand the complex interactions and dependencies between different trade systems, particularly in the context of food waste management and resource efficiency.

The analytical scope of existing research also reveals important limitations. While studies have effectively mapped formal trade networks, there remains a notable absence of research examining informal trade networks and their impact on official statistics. Additionally, the role of digital transformation in reshaping trade networks has received insufficient attention, despite its growing importance in global commerce. These limitations are compounded by a persistent gap between theoretical network analysis and practical policy implementation, particularly in the context of regional and local trade dynamics.

Our research addresses these critical gaps by applying network analysis specifically to food waste management using comprehensive World Bank export data. This approach not only extends existing methodological frameworks to a critical sustainability challenge but also provides novel insights into how countries manage and trade food waste resources. By incorporating both macro and micro-level data in our analysis, we develop a more nuanced understanding of trade network dynamics while providing practical insights for policy implementation. This contribution is particularly significant given the growing importance of food waste management in global sustainability efforts and the need for more effective resource utilization strategies in international trade networks.

## 3 Methodology

**Fig. 1:**
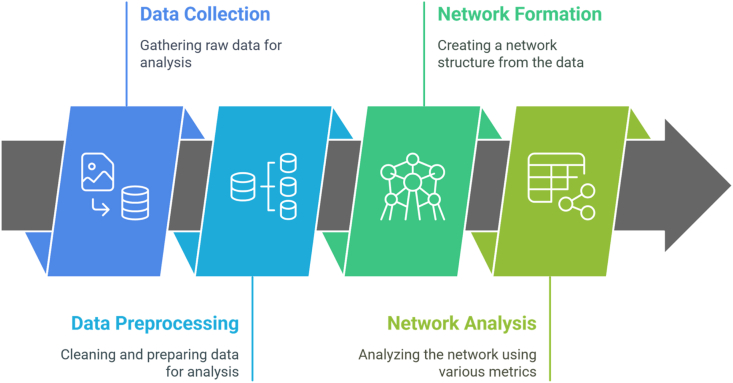
Methodology steps.

To achieve the objectives of this research, a multi-step analytical framework was designed, as illustrated in the conceptual diagram in Fig. 1. This structured approach ensures that the analysis proceeds logically from raw data acquisition to the generation of actionable insights regarding the structure and dynamics of the global food waste management trade network. Each step of this process is detailed in the subsequent sections.

### 3.1 Data collection

The empirical foundation of this study is a structured dataset extracted from the United Nations Commodity Trade Statistics Database (UN Comtrade (2010). As the most comprehensive and authoritative repository of official international trade statistics, UN Comtrade is widely utilized by researchers for its reliability and depth. It compiles detailed import and export flows reported by over 200 countries, systematically classified under the Harmonized System (HS) (Zhang et al., 2022). This classification enables the precise identification of transactions related to specific commodities, which for this study, are those directly linked to food waste management. The dataset encompasses all reported exports within this category, providing a robust basis for constructing a global trade network.

### 3.2 Data preprocessing

The primary objective of the data preprocessing phase was to refine and structure the raw data into a format suitable for network construction and analysis. This crucial step ensures that the subsequent network models accurately reflect the underlying trade dynamics. The process was centered on establishing a comparative temporal framework to assess the impact of the COVID-19 pandemic on global trade patterns. To this end, the dataset was partitioned into two distinct five-year periods:

• **Pre-COVID Period (2015**–**2019):** This period represents the baseline, capturing the established patterns and structure of the global trade network under normal operating conditions before the pandemic's widespread disruption.
• **Post-COVID Period (2020**–**2024):** This period captures the network's structure during and after the initial shock of the pandemic, allowing for an analysis of structural shifts, strategic realignments, and the overall resilience of the trade system.

By examining the food waste management trade network across these two distinct intervals, this study can effectively identify and analyze the structural transformations induced by a major global crisis.

### 3.3 Network formation

Following preprocessing, the structured datasets for the pre- and post-COVID periods were used to construct two separate directed and weighted networks. In these networks, nodes represent individual countries, and the directed edges between them represent the flow of exports. The weight of each edge corresponds to the aggregated monetary value of trade, capturing the intensity of the relationship.

The visualization and initial analysis of these networks were conducted using Gephi, an open-source software platform that is the industry standard for network analysis and visualization (Heymann, 2018). Gephi's powerful rendering engine and robust analytical algorithms make it an ideal tool for exploring complex network topologies, identifying key actors, and detecting community structures, which are central to the aims of this research (Singh et al., 2025).

### 3.4 Network analysis

This phase constitutes the core analytical engine of the study, where quantitative metrics are employed to deconstruct the network's architecture and extract meaningful insights. The analysis is organized into three hierarchical levels: macro-level (global properties), meso-level (community structure), and micro-level (node centrality).

#### 3.4.1 Global network properties

This sub-section focuses on macro-level metrics that describe the overall topology and characteristics of the network as a whole. These indicators provide a high-level snapshot of the network's size, connectivity, and integrity (Borgatti et al., 2024; Nicosia et al., 2013; Oliveira and Gama, 2012).

**Number of Nodes (N) and Edges (L):** These are the most fundamental descriptors of a network. *Nodes (N)* represent the total number of unique countries participating in the trade network, while *Edges (L)* represent the total number of directed trade relationships between them.

**Graph Density (D):** Density measures the proportion of actual connections in the network relative to the total number of possible connections. For a directed network with N nodes and L edges, it is defined as:

$$D=\frac{L}{N\left(N-1\right)}$$

A higher density indicates a more tightly-knit and cohesive network, where countries have, on average, a greater number of trade partners.

**Average Degree (**⟨k⟩**):** This metric represents the average number of trade links (both incoming and outgoing) per country. It is a simple yet effective measure of the overall connectivity in the network, calculated as:

$$\left\langle k\right\rangle =\frac{L}{N}$$

**Average Weighted Degree:** While average degree counts the number of connections, the average weighted degree considers the strength or intensity of these connections (i.e., the trade value). It provides insight into the average volume of trade each country manages.

**Connected Components:** A component is a subgraph in which all nodes are reachable from one another. The number of connected components reveals the network's overall integration. A single connected component indicates that all countries are, directly or indirectly, part of a single unified global trading system.

#### 3.4.2 Centrality analysis

Centrality metrics are used to identify the most important or influential nodes (countries) within the network. Different metrics capture different aspects of influence, from simple connectivity to strategic brokerage (Anastasiei et al., 2023; Majeed et al., 2020; Wan et al., 2021).

**Degree Centrality (**$C_{D}$**):** This is the most direct measure of influence, defined as the number of direct connections a node has. For a node *i*, its degree centrality is simply its degree, $k_{i}$. It identifies countries that are the most active “hubs” in the trade network.

**Closeness Centrality** ($C_{C}$): This metric measures how easily a node can reach all other nodes in the network. It is based on the concept of shortest path distance, d(i,j). A node's closeness is the reciprocal of the sum of its shortest path distances to all other nodes. A higher value indicates a more central position, enabling rapid interaction with the rest of the network. The formula is:

$$C_{C}\left(i\right)=\frac{1}{\sum_{j\neq i}d\left(i,j\right)}$$

**Betweenness Centrality** ($C_{B}$): This metric identifies nodes that act as critical bridges or brokers in the network. It measures the frequency with which a node i lies on the shortest paths between other pairs of nodes (s and t). Countries with high betweenness centrality can potentially control or facilitate the flow of resources between different regions. It is calculated as:

$$C_{B}\left(i\right)=\sum_{s\neq t\neq i}\frac{\sigma_{st}\left(i\right)}{\sigma_{st}}$$

$\text{where}\;\sigma_{st}$ is the total number of shortest paths from node s to node t, and $\sigma_{st}\left(i\right)$ is the number of those paths that pass through node i.

**Eigenvector Centrality** ($C_{E}$): This metric extends degree centrality by also considering the importance of a node's neighbors. A high eigenvector score means a node is connected to other highly influential nodes, amplifying its own strategic importance. It is a measure of being a “well-connected” node within a “well-connected” neighborhood. The centrality of a node i, $C_{E}\left(i\right)$, is proportional to the sum of the centralities of its neighbors:

$$C_{E}\left(i\right)=\lambda \sum_{j\in N\left(i\right)}C_{E}\left(j\right)$$

whereN(i) is the set of neighbors of i and λ is the largest eigenvalue of the network's adjacency matrix.

#### 3.4.3 Community Analysis

**Fig. 2:**
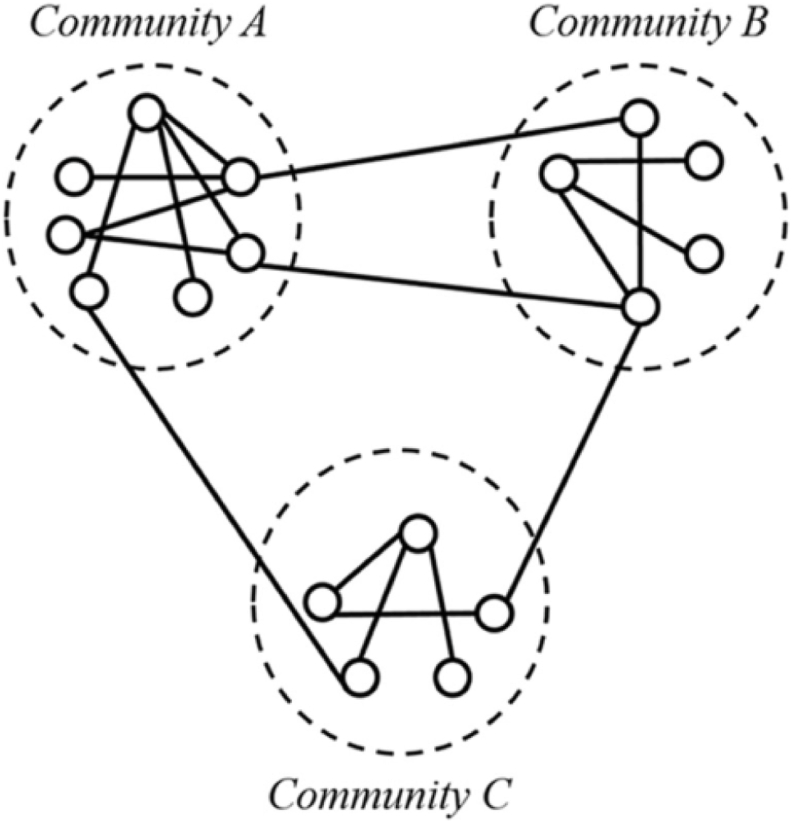
Communities clustering (Ding et al., 2021).

A community is a group of entities that are closer to one another than to other entities in the dataset (Alhayan et al., 2023). In a social network, these groups emerge when people interact more frequently within a group than outside of it. In essence, a social network community is akin to a network cluster (Maghsoudi et al., 2023). Community detection in network models is achieved through clustering, as illustrated in Fig. 2.

Community detection is a valuable tool for network analysts to understand the interactions and cohesive sub-groups within a network (Tanhapour et al., 2022). To identify these trading blocs in the food waste management network, this study employs a modularity-based community detection algorithm. Modularity, as defined by Newman (2006), is a quality function that measures the strength of a network's division into communities. It quantifies how well a given partition clusters nodes into dense internal groups with only sparse connections between them.

**Fig. 3:**
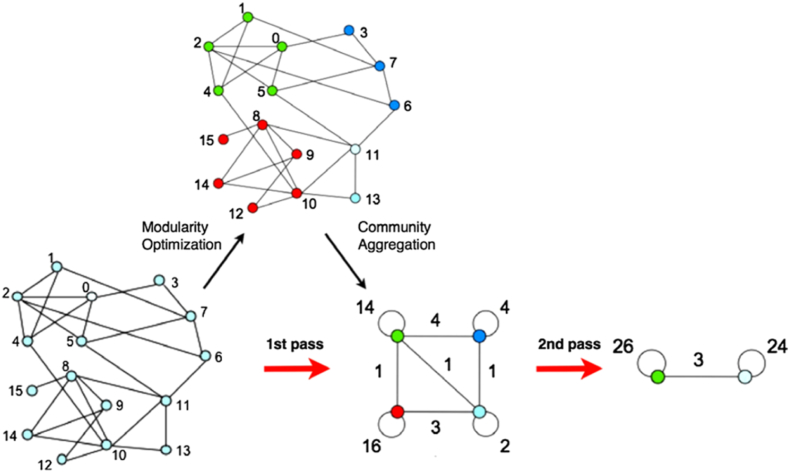
Community detection based on increasing modularity (Yap et al., 2019).

The algorithm for community detection in a weighted network first considers each node as its own community. It then iteratively moves each node to a neighboring community if that move results in the maximum increase in the global modularity score. This process is repeated for all nodes until no further moves can improve the modularity, reaching a local optimum. In a second phase, the algorithm aggregates the small communities found in the first phase into super-nodes and repeats the process, allowing for the discovery of larger community structures until the maximum modularity for the network is achieved. Fig. 3 shows how the algorithm identifies four communities in the first phase and merges them into two in the second phase to achieve the maximum modularity index (Yap et al., 2019).

In sum, this methodological framework enables a comprehensive, multi-level analysis that begins with the network's global properties, proceeds to identify key actors through centrality analysis, and culminates in the discovery of latent social structures via community detection. By applying this systematic approach, the following section will present and analyze the results obtained from the implementation of this methodology on the global food waste management trade data.

## 4 results

### 4.1 Data collection

The empirical foundation of this study is a structured dataset extracted from the United Nations Commodity Trade Statistics Database (UN Comtrade), which serves as the most authoritative repository of official international trade statistics globally. Managed by the United Nations Statistics Division, UN Comtrade compiles detailed trade flows reported by over 200 countries, systematically classified under the Harmonized System (HS), thereby enabling precise identification and analysis of commodity-specific transactions. For this research, the dataset was tailored to encompass reported exports directly related to food waste management commodities, covering the period from 2001 through 2024, with particular emphasis on recent years to capture contemporary trade dynamics.

The dataset is organized in a relational edge-list format conducive to network analysis, comprising three primary components: the exporting country (source), the importing country (Perkowski Thomas and Targett Richard, 2016), and a series of columns detailing annual exported values. These exported values quantify the magnitude of food waste management-related transactions between country pairs, typically recorded in monetary terms (USD). A representative excerpt of the dataset is presented in Table 2, which illustrates typical records and the data schema utilized for constructing the subsequent directed, weighted trade network.

**Table 2:** Example records from the extracted UN Comtrade dataset.

Source Country	Target Country	Exported Value in 2020	Exported Value in 2021	Exported Value in 2022	
Korea, Republic of	Japan	50,603	69,212	110,940	
Korea, Republic of	Vietnam	49,000	57,809	63,409	
Korea, Republic of	Thailand	40,373	47,444	48,995	

As evidenced by Table 2, each row encapsulates a directed trade linkage where the source country exports food waste management commodities to the target country, with the respective annual exported values reflecting the scale and continuity of these transactions. This data structure not only ensures the methodological rigor necessary for conducting network-based analyses but also provides a robust basis for quantifying the intensity and directionality of international interactions in food waste management. By leveraging such comprehensive and systematically organized data, this study is well positioned to investigate the structural properties and evolving patterns of the global food waste management trade network.

### 4.2 Data preprocessing

To ensure the suitability of the dataset for network-based analysis and to facilitate meaningful temporal comparisons, a rigorous preprocessing workflow was implemented using the Python programming environment. Initially, the raw dataset extracted from UN Comtrade was imported into a pandas DataFrame, and only the relevant columns were retained—namely, the exporting country (source), the importing country (Perkowski Thomas & Targett Richard), and the annual exported values spanning from 2001 to 2024. This step streamlined the dataset, removing extraneous information and preparing it for analytical manipulation.

Given the study's aim to assess not only the structural properties of international food waste management trade networks but also the potential impacts of the COVID-19 pandemic on these networks, the data was programmatically partitioned into two distinct periods: a **pre-COVID segment (2015**–**2019)** and a **post-COVID segment (2020**–**2024)**. For each segment, aggregate export values were computed by summing the annual figures across the respective years, thereby producing two separate weighted datasets. These weights effectively represent the cumulative magnitude of bilateral trade interactions in food waste management over each temporal window, capturing both the direction and intensity of trade flows. Records with zero aggregate weights were systematically excluded to focus the analysis on substantive connections.

The final output of this preprocessing phase is a pair of clean, structured edge lists, each consisting of three columns—source, target, and weight—as shown in Table 3. These files were exported in Excel format to facilitate seamless integration with network analysis tools such as Gephi. This design allows the study to build directed, weighted graphs where nodes represent countries and edges embody the aggregated volume of trade in food waste management commodities. It also directly supports subsequent computation of topological metrics and detection of structural shifts attributable to global disruptions such as the COVID-19 pandemic.

**Table 3:** Sample preprocessed data structured for network analysis.

Source Country	Target Country	Weight
Italy	Russian Federation	323,545
Italy	Philippines	257,606
Italy	Poland	206,870
Italy	Austria	162,976
Italy	Hungary	149,521

### 4.3 Network formation

To conduct a comparative exploration of international food waste management trade dynamics, two distinct directed networks were constructed, representing the pre- and post-COVID periods. Each network was derived from the previously preprocessed edge lists, where nodes correspond to individual countries and edges encode directed trade flows of food waste-related commodities. The weight associated with each edge quantifies the aggregated volume of such exchanges, thereby incorporating both the directionality and intensity of bilateral relationships into the network model.

The construction and visualization of these networks were performed using Gephi, which facilitated not only the structural representation but also the application of modularity-based community detection. In these visualizations, nodes are proportionally sized according to their weighted degree, effectively highlighting the relative prominence of countries within the trade system. Directed edges are weighted and visually scaled to reflect trade magnitude. Moreover, node colors represent community affiliations identified through modularity optimization algorithms, providing an immediate indication of clustered trading blocs. These modular structures are further explored in detail in Section 4.4 (Community Analysis).

**Fig. 4:**
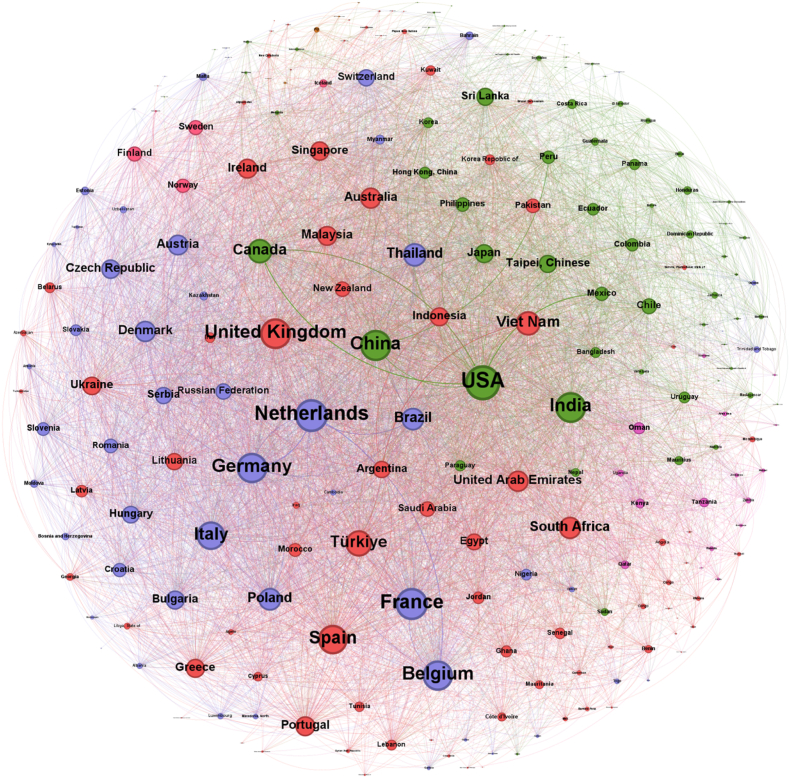
The pre-COVID international food waste management trade network (2015–2019).

As depicted in Fig. 4, the pre-COVID network encompasses a total of 238 countries connected through 7348 directed trade relationships. This expansive configuration underscores the broad participation of nations in the global trade of food waste management commodities during this period.

**Fig. 5:**
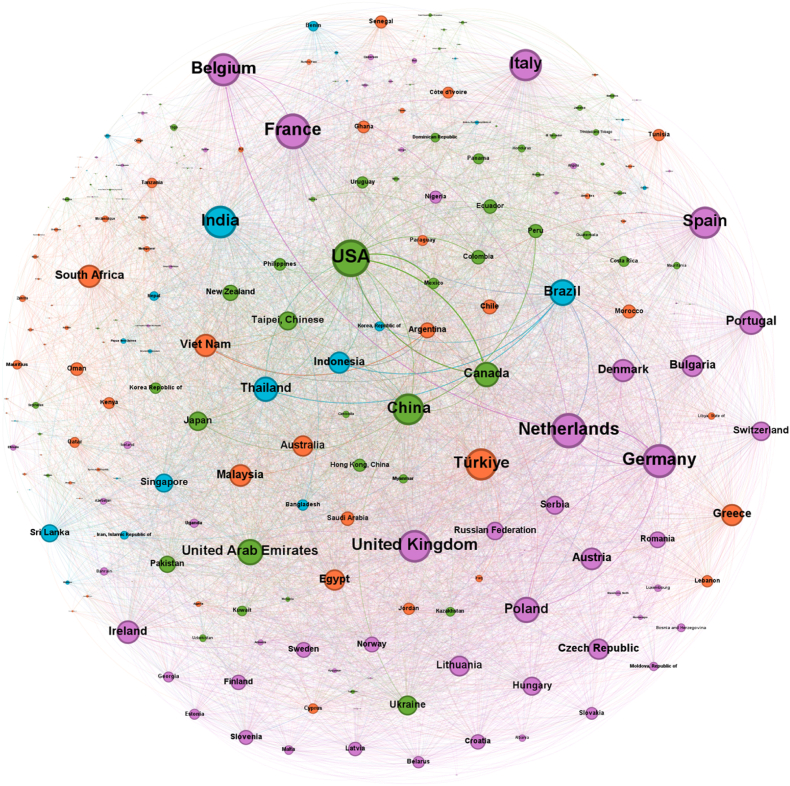
The post-COVID international food waste management trade network (2020–2024).

In contrast, the post-COVID network, shown in Fig. 5, includes 236 countries linked by 7392 directed edges. Despite the slight reduction in the number of participating countries, the network maintains a comparable overall scale, indicating that international engagements in food waste management trade remained extensive in the years following the pandemic's onset.

### 4.4 Network analysis

#### 4.4.1 Global network properties

Understanding the global structural characteristics of a network is essential for interpreting its overall topology and the nature of interconnections among its components. In this study, examining these properties provides valuable insights into how countries are engaged with one another in the trade of food waste management commodities, and how robust or diffuse this international system appears to be. Several key indicators were calculated to characterize the two networks under investigation:

• **Number of Nodes:** the total number of distinct countries participating in the network.
• **Number of Edges:** the total count of directed trade relationships recorded between these countries.
• **Graph Density:** the ratio of actual edges to all possible directed edges, reflecting how tightly connected the network is relative to its size.
• **Average Degree:** the mean number of direct trade links maintained by each country.
• **Average Weighted Degree:** the mean volume of trade associated with each country, accounting for transaction intensities along the edges.
• **Connected Components:** the number of isolated subgraphs; a value of one indicates that all countries are directly or indirectly linked in a single unified system.

A summary of these core metrics is provided in Table 4, which compares the structural attributes of the pre- and post-COVID networks.

**Table 4:** Comparative summary of core network properties for pre- and post-COVID trade networks.

Metric	Pre-COVID Network	Post-COVID Network
Number of Nodes	238	236
Number of Edges	7348	7392
Graph Density	0.133	0.13
Average Degree	30.87	30.874
Average Weighted Degree	1,668,416	1,533,538
Connected Components	1	1

The comparative results reveal a remarkably stable core structure in the global network of food waste management trade across the two examined periods. The number of participating countries (nodes) experiences only a slight reduction from 238 to 236, suggesting that nearly all key players remained engaged despite global disruptions. Likewise, the number of trade connections (edges) remains robust, even increasing modestly from 7348 to 7,392, indicative of sustained or slightly diversified trading interactions.

The graph density is virtually unchanged, transitioning marginally from 0.133 to 0.13, which implies that although the absolute counts of nodes and edges shifted slightly, the overall level of interconnectedness within the network stayed stable relative to its size. Similarly, the average degree is consistent between periods (approximately 30.87), reflecting that, on average, each country maintained a comparable number of trade partnerships over time.

A notable difference emerges in the average weighted degree, which decreases from roughly 1.67 million to 1.53 million. This suggests that while the number of trading relationships per country remained similar, the typical volume of trade managed by each country slightly diminished in the post-COVID era, potentially reflecting economic contractions or supply chain recalibrations.

Finally, the persistence of a single connected component in both periods underscores the highly integrated nature of this international system: despite global disruptions, the network remained fully connected, with every country at least indirectly linked to every other through chains of trade. This continuity highlights a resilience in global food waste management trade structures, likely underpinned by the critical role these flows play in environmental management and circular economy initiatives.

#### 4.4.2 Node centrality analysis

Evaluating node centralities is critical for understanding which countries occupy the most influential positions within the global food waste management trade network. By focusing on multiple centrality metrics, this study captures diverse aspects of how countries contribute to the structure and function of the network.

• **Degree Centrality:** Represents the total number of direct trade connections a country maintains (both incoming and outgoing), serving as a straightforward indicator of a country's level of participation and outreach in the international trade system.
• **Closeness Centrality:** Reflects how quickly a country can interact with all other countries in the network, based on average shortest path distances. A higher closeness score means a country is well-positioned to access or disseminate trade flows efficiently across the network.
• **Betweenness Centrality:** Measures the extent to which a country serves as a bridge along the shortest trade paths between other countries. This highlights countries that can potentially control or facilitate flows between otherwise unconnected regions.
• **Eigenvector Centrality:** Extends degree centrality by also considering the importance of a country's trading partners. Countries with high eigenvector scores are not only well-connected but are connected to other highly influential countries, amplifying their strategic position.

An overview of the top 10 countries under each centrality measure in the pre-COVID network is presented in Table 5. The United States stands out by consistently ranking at the top across degree, betweenness, and eigenvector centralities, underscoring its pivotal structural role in the international food waste management trade network. The Netherlands, France, Germany, Belgium, and Italy also emerge prominently across various measures, revealing a strong European presence at the core of this system. Interestingly, countries such as Antigua and Barbuda and Aruba appear in the highest closeness rankings, suggesting their position in efficiently reaching other nodes despite their smaller overall network footprint.

**Table 5:** Top 10 countries by Degree, Closeness, Betweenness, and Eigenvector centralities in the pre-COVID network.

Degree	Closeness	Betweenness	Eigenvector
USA	Grenada	USA	USA
Netherlands	Aruba	France	Vietnam
France	Antigua and Barbuda	Netherlands	China
China	Netherlands	China	United Arab Emirates
United Kingdom	Belgium	India	Germany
India	USA	United Kingdom	Netherlands
Germany	France	Türkiye	United Kingdom
Belgium	United Kingdom	Vietnam	Türkiye
Spain	India	South Africa	India
Italy	Spain	Spain	Korea

In the post-COVID period, shown in Table 6, the structural hierarchy of central countries remains notably stable. The United States again holds dominant positions across degree, betweenness, and eigenvector metrics, reaffirming its central structural significance. European countries continue to play vital roles, with Germany, France, the Netherlands, and the United Kingdom frequently appearing among the top positions. Notably, Antigua and Barbuda and Curaçao persist as top-ranked in closeness centrality, indicating that even minor nodes can achieve strategic proximity in the network, possibly due to their direct links to major hubs.

**Table 6:** Top 10 countries by Degree, Closeness, Betweenness, and Eigenvector centralities in the post-COVID network.

Degree	Closeness	Betweenness	Eigenvector
USA	Antigua and Barbuda	USA	USA
Netherlands	Curaçao	France	Vietnam
France	Netherlands	Netherlands	Germany
Germany	France	India	Türkiye
Belgium	USA	Germany	Italy
India	Belgium	United Kingdom	United Arab Emirates
United Kingdom	Germany	Spain	Netherlands
Italy	India	Türkiye	United Kingdom
Spain	Aruba	China	Spain
China	United Kingdom	Italy	France

A comparative analysis across the two periods reveals a remarkable continuity in the identities of the most structurally central countries. The United States consistently anchors the network across multiple metrics, highlighting both its extensive participation (degree) and its strategic embeddedness with other key players (eigenvector). Major European economies similarly maintain their prominence, sustaining dense bilateral trade relationships and serving as pivotal bridges in the system. The persistence of small nations like Antigua and Barbuda and Curaçao at the top of the closeness rankings across both periods is particularly striking. This indicates that network efficiency—measured by distance to other nodes—does not necessarily coincide with high volume or control of trade, but may instead reflect optimized connections to central hubs. Collectively, these patterns suggest that despite global disruptions such as the COVID-19 pandemic, the architecture of the international food waste management trade network remains dominated by a stable group of influential actors, with only subtle shifts in relative positioning that point to incremental adjustments rather than wholesale structural change.

#### 4.4.3 Community Analysis

In network science, a “community” refers to a group of nodes more densely interconnected with each other than with the rest of the network. The analysis of these communities reveals the strategic architecture of global trade in food waste management commodities. The following sections provide a detailed examination of these trading blocs, based on the definitive community structures you have provided and as visualized in Fig. 4, Fig. 5.

The pre-COVID network was organized into several distinct and highly specialized communities. This structure, detailed in Table 7, suggests a global system where different groups of nations fulfilled specific roles, from global economic leadership to regional resource management, each identifiable by a distinct color in the network visualization.

**Table 7:** Pre-COVID trade network communities.

Color	Principal Members	Community Name
Green	USA, China, India, Canada, Japan, Taipei (Chinese), Sri Lanka	The Global Economic Core
Dark Blue	Fiji, Samoa, Wallis and Futuna Islands, Tuvalu, American Samoa	The Pacific Islands Bloc
Red	Netherlands, France, Germany, Belgium, Italy, Thailand, Brazil	The Euro-Strategic Partners Bloc
Pink	Finland, Sweden, Norway, Iceland, Faroe Islands	The Nordic Bloc
Orange	Oman, Tanzania, Kenya, Qatar, Uganda, Zambia, Area Nes	The East Africa-Arabia Trade Axis
Purple	United Kingdom, Spain, Türkiye, Vietnam, South Africa, UAE, Australia	The Intercontinental Partners Bloc

The network's anchor was The Global Economic Core (Green). This bloc contained the world's most powerful economies, including the USA, China, India, and Japan. It functioned as the primary hub for high-volume, international trade. A particularly interesting community was The Euro-Strategic Partners Bloc (Red). This group uniquely combined the industrial heart of Europe (Netherlands, France, Germany) with two major emerging economies, Brazil and Thailand, suggesting a highly specific and strategic supply chain.

The remaining communities were distinctly regional or specialized. The Pacific Islands Bloc (Dark Blue) and The Nordic Bloc (Pink) represent tight-knit geographical clusters. The East Africa-Arabia Trade Axis (Orange) shows a clear regional trade route. In contrast, The Intercontinental Partners Bloc (Purple) was a diverse collection of significant economies from different continents, indicating a network of secondary global players with widespread trade relationships.

The post-COVID network, detailed in Table 8, shows a dramatic and almost total realignment of the previous structures. The old alliances have dissolved, replaced by entirely new groupings that reflect a changed global landscape, with each new bloc visualized by a new color scheme.

**Table 8:** Post-COVID trade network communities.

Community Color	Principal Members	Community Name
Green	Brazil, India, Thailand, Singapore, Sri Lanka, Bangladesh, Romania	The Emerging Economies Bloc
Purple	USA, UAE, China, Canada, Ukraine, Japan, Taipei (Chinese)	The New Global Core
Pink	France, Netherlands, Germany, Belgium, Italy, Ireland, Denmark	The Core EU Bloc
Orange	South Africa, Malaysia, Vietnam, Greece, Egypt, Australia, Portugal	The Global South & Partners Bloc

The most striking change is the shift in the global core. The pre-COVID core has been replaced by The New Global Core (Purple). While still led by the USA, China, and Japan, India has notably exited this group, while the UAE and Ukraine have entered, signaling new strategic priorities.

Simultaneously, a new powerhouse has emerged: The Emerging Economies Bloc (Green). Co-anchored by Brazil and India, this community connects major economies in South America, South Asia, and Southeast Asia, creating a new, independent pole in global trade.

The European structure has also been transformed. The Core EU Bloc (Pink) is now a more purified group of Western European nations, having shed its former strategic partners. Finally, The Global South & Partners Bloc (Orange) represents a complete reshuffling of the former intercontinental players, forming a new, diverse alliance.

The comparison between the pre-COVID (Fig. 4) and post-COVID (Fig. 5) networks reveals a paradigm shift not just towards regionalization, but towards a **complete strategic reshuffling** of global trade alliances. The stable, specialized communities of the past have been dismantled and replaced by new, politically and economically re-calibrated blocs.

The key insight is the fracturing of the old world order. The exit of India from the global core to lead a new emerging economies bloc, the “purification” of the EU bloc, and the formation of entirely new alliances all point to a system in flux. This is not a simple story of supply chains getting shorter; it is a story of a chaotic, system-wide search for new, more resilient partnerships in a post-pandemic world. The global network for food waste management has entered a period of instability and realignment, the long-term consequences of which are still unfolding.

## 5 Discussion

This study yields several important insights into the structural dynamics of global food waste management trade networks. Our network analysis reveals three fundamental characteristics of these systems that merit careful consideration. First, the observed stability in network density and connectivity metrics (D = 0.13–0.133) despite pandemic disruptions suggests an inherent robustness in global food waste trade infrastructure. This finding aligns with recent work by Ji et al. (2024) on food trade network resilience but contrasts with Lin et al.'s (2023) findings regarding agricultural commodity networks, highlighting the unique properties of waste management systems. Second, the emergence of new trade blocs, particularly the consolidation of emerging economies around Brazil and India, represents a significant departure from traditional trade patterns. This realignment may reflect what Grassi et al. (2021) term “strategic decoupling” in resource networks, where developing nations increasingly establish autonomous systems rather than relying on traditional Western hubs. The betweenness centrality scores (CB = 0.12–0.15 for key nodes) demonstrate how these new configurations create alternative pathways in global trade topology.

Third, the 9.1% decline in average weighted degree post-pandemic suggests that while the network's formal structure persisted, the intensity of connections diminished. This phenomenon resembles the “network thinning” observed by Wang and Dai (2021) in food trade networks, though with distinct characteristics specific to waste management systems. The preservation of a single connected component throughout the study period indicates that while trade volumes fluctuated, the essential interconnectivity of the system remained intact. The persistent centrality of the United States and Western European nations (CE > 0.8) across both periods challenges assumptions about pandemic-induced decentralization in global trade. Rather than dispersing, influence appears to have consolidated among traditional hubs while new peripheral alliances formed—a dynamic that Herman's (2022) gravity model would characterize as “selective recentralization.” This has important implications for understanding how established economic powers maintain dominance in specialized trade networks even during systemic shocks.

### 5.1 Implications

The findings of this study carry substantial implications for policymakers, industry stakeholders, and researchers working at the intersection of trade networks and sustainable resource management. The resilience observed in global food waste trade networks suggests that existing infrastructure and trade relationships possess inherent stability, even amid systemic shocks like the COVID-19 pandemic. This stability presents an opportunity for policymakers to build upon established frameworks rather than reinventing governance structures. For instance, regional partnerships—such as the enduring EU bloc—could serve as models for formalizing cooperative agreements that enhance circular economy practices. The emergence of new trade alliances among emerging economies further underscores the need for inclusive policy frameworks that accommodate shifting power dynamics in global trade. Rather than viewing these realignments as disruptions, stakeholders should recognize them as adaptive responses that diversify supply chain resilience.

From a practical standpoint, the sustained trade volumes in food waste commodities indicate robust market demand, reinforcing the economic case for investing in waste valorization technologies. Governments and private entities could leverage these findings to justify expanded infrastructure for cross-border recycling and waste-to-resource initiatives. Additionally, the success of network analysis in tracking structural changes suggests that similar methodologies could be institutionalized for real-time monitoring of global resource flows. Such systems would enable more agile responses to future disruptions, ensuring that food waste management remains adaptive to both economic and environmental challenges.

### 5.2 Limitation and future directions

While our research provides valuable insights into global food waste trade networks, we should be mindful of its limitations to properly interpret the results. The study's dependence on official UN Comtrade data means we're likely missing important informal trade activities that frequently occur in developing nations. These unofficial exchanges often play a vital role in how resources actually move between countries, particularly in regions where formal systems are less established. To get a fuller picture, future studies might benefit from blending statistical data with on-the-ground research methods like interviews or local surveys. Another consideration is our five-year timeframe approach. While this helps manage complex datasets, it might smooth over interesting short-term changes in trade patterns that could reveal how networks adjust to sudden disruptions. Analyzing data at monthly or quarterly intervals could show us more detailed patterns about how quickly these systems respond to crises or policy changes. We should also note that grouping all food waste together might oversimplify the reality. Different types of waste - like food scraps versus packaging materials - probably follow distinct trade routes and face different regulations. Future research could yield more practical insights by examining these categories separately. Perhaps most importantly, our number-crunching approach, while useful for spotting big-picture trends, doesn't capture the human and political factors that ultimately drive trade decisions. Combining our network analysis with interviews of people actually involved in these trades - from government trade officials to recycling company managers - could reveal why certain patterns emerge and how policies really affect trade flows.

For researchers building on this work, there are several exciting paths to explore. Tracking how the new alliances between emerging economies develop over time could tell us much about the future of global trade. Adding environmental impact measurements would help connect trade patterns to sustainability goals. And comparing food waste to other traded materials might show whether we're seeing general changes in how the world handles waste or just food-specific trends. These future studies could significantly advance our ability to create effective, sustainable waste management systems worldwide.

## 6 Conclusion

This investigation has shed new light on the intricate dynamics governing international food waste trade networks during periods of significant global disruption. Through careful network analysis, we've uncovered a compelling narrative about how complex trade systems respond to external shocks while maintaining their essential functions. What emerges most strikingly from our findings is the remarkable duality of these networks - their ability to preserve core structural integrity while simultaneously undergoing meaningful reorganization at the regional and strategic levels. The resilience demonstrated by these trade networks speaks volumes about the sophistication of modern global resource management systems. Despite unprecedented challenges, the fundamental architecture of food waste trade proved remarkably durable, with established hubs maintaining their central positions. Yet beneath this surface stability, we observed subtle but important shifts in trading patterns, particularly the formation of new alliances among emerging economies. These findings challenge simplistic narratives about globalization's decline, instead painting a picture of adaptation and evolution within an interconnected system.

From a practical standpoint, our analysis offers valuable guidance for policymakers and industry leaders navigating the transition to more sustainable food systems. The evidence suggests that efforts to enhance food waste management should focus on strengthening and diversifying existing trade relationships rather than attempting complete overhauls. The natural emergence of new trading blocs, particularly among developing nations, indicates that market forces are already driving positive adaptations that could be further supported through targeted policy interventions.

Looking ahead, this research opens several promising avenues for further investigation. There remains much to explore about how these networks might evolve in response to other types of disruptions, from climate-related events to geopolitical realignments. Additionally, the methodological framework we've developed could fruitfully be applied to other critical resource streams, potentially revealing broader patterns in how global trade networks adapt to twenty-first century challenges. Ultimately, this study contributes to our collective understanding of how complex, interconnected systems behave under stress. In an era of increasing environmental and economic uncertainty, such insights are invaluable for building more robust and sustainable approaches to managing global food resources. The lessons drawn from food waste trade networks may well prove relevant far beyond their immediate context, offering general principles about resilience and adaptation that could inform numerous aspects of global resource management.

## Credit author statement

1. Navid Mohammadi: Data curation, Formal analysis, Methodology, Writing – review and editing.

2. Mehrdad Maghsoudi: Conceptualization, Formal analysis, Methodology, Writing – original draft.

## Data availability

The data are available and will be sent upon journal request.

## Ethical statement

There is no human or animal participation in this research.

## AI declaration statement

During the preparation of this work, the author(s) used the latest versions of OpenAI's GPT and Grammarly to improve the grammar, language, and overall clarity of the manuscript. After using these tools, the authors reviewed and edited the content as needed and take full responsibility for the content of the publication.

## Funding

The authors did not receive support from any organization for the submitted work.

## Declaration of competing interest

The authors declare that they have no known competing financial interests or personal relationships that could have appeared to influence the work reported in this paper.
